# Single therapeutic dose of an antiviral UL29 siRNA swarm diminishes symptoms and viral load of mice infected intranasally with HSV‐1

**DOI:** 10.1002/SMMD.20230009

**Published:** 2023-05-10

**Authors:** Tuomas Lasanen, Fanny Frejborg, Liisa M. Lund, Marie C. Nyman, Julius Orpana, Huda Habib, Salla Alaollitervo, Alesia A. Levanova, Minna M. Poranen, Veijo Hukkanen, Kiira Kalke

**Affiliations:** ^1^ Faculty of Medicine Institute of Biomedicine University of Turku Turku Finland; ^2^ Faculty of Science and Engineering Pharmaceutical Sciences Laboratory Åbo Akademi University Turku Finland; ^3^ Molecular and Integrative Biosciences Research Programme Biological and Environmental Sciences University of Helsinki Helsinki Finland

**Keywords:** antiviral, herpes simplex virus, infection models, intranasal, RNA interference, siRNA swarms

## Abstract

Herpes simplex virus type 1 (HSV‐1) is a human pathogen that causes recurrent infections. Acyclovir‐resistant strains exist and can cause severe complications, which are potentially untreatable with current therapies. We have developed siRNA swarms that target a 653 base pair long region of the essential HSV gene *UL29*. As per our previous results, the anti‐UL29 siRNA swarm effectively inhibits the replication of circulating HSV strains and acyclovir‐resistant HSV strains in vitro, while displaying a good safety profile. We investigated a single intranasal therapeutic dose of a siRNA swarm in mice, which were first inoculated intranasally with HSV‐1 and given treatment 4 h later. We utilized a luciferase‐expressing HSV‐1 strain, which enabled daily follow‐up of infection with in vivo imaging. Our results show that a single dose of a UL29‐targeted siRNA swarm can inhibit the replication of HSV‐1 in orofacial tissue, which was reflected in ex vivo HSV titers and HSV DNA copy numbers as well as by a decrease in a luciferase‐derived signal. Furthermore, the treatment had a tendency to protect mice from severe clinical symptoms and delay the onset of the symptoms. These results support the development of antiviral siRNA swarms as a novel treatment for HSV‐1 infections.

## Graphical abstract

An antiviral siRNA swarm targeting the viral *UL29* gene proved effective against HSV‐1 upon topical administration in an intranasal model of infection. The results were confirmed by in vivo imaging, enabled by the luciferase transgene of the virus, as well as by PCR for viral genomes and plaque titration for the live virus. In comparison with placebo treatment (a nonspecific siRNA swarm), the UL29 siRNA swarm delayed onset of symptoms as well as decreased local viral load and replication.

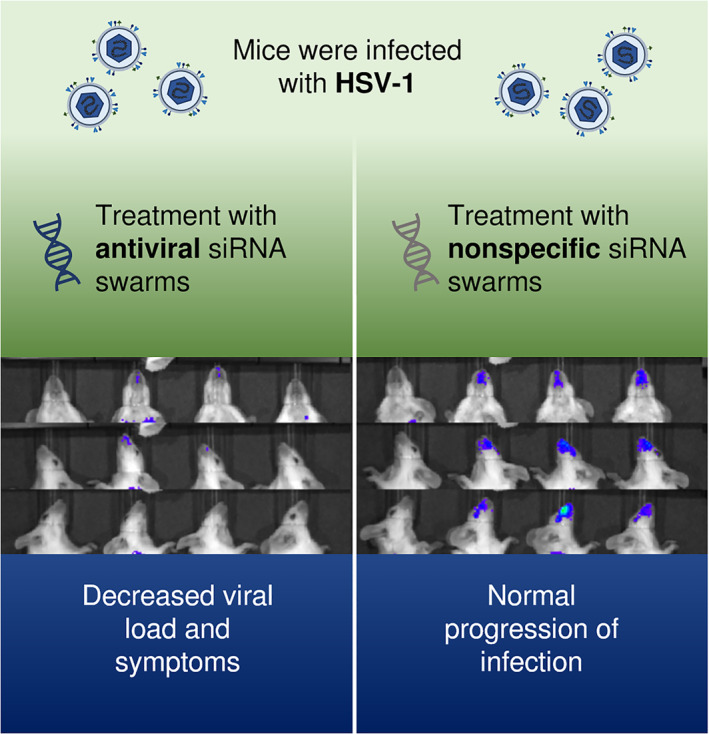

**Key points**

- A single therapeutic intranasal dose of UL29 siRNA swarm is antiviral in vivo.
- The UL29 siRNA swarm inhibits viral replication and decreases viral load in mice.
- The UL29 siRNA swarm has a tendency to delay the onset of clinical symptoms in mice and to protect them from severe clinical symptoms.
- The antiviral efficacy of the siRNA swarm was visualized utilizing a luciferase transgene HSV as treatment target.

## 1 INTRODUCTION

Herpes simplex viruses (HSV) are common human pathogens that establish latency in the nervous system. The most common manifestations of HSV‐1 infections are recurrent orofacial lesions, though the infection may cause rare, but severe, encephalitis or keratitis. While acyclovir and its structural analogs are well‐established treatments for active infection, drug‐resistant strains exist and cause severe complications, especially in the immunocompromised population.^1^ To answer this unmet medical need, we have developed a novel therapy: antiviral siRNA swarms. Antiviral siRNA swarms differ from regular siRNA products by their enzymatic synthesis^2^ and by their longer target sequence, which has been even up to 3500 base pairs.^3^ The individual siRNAs of the siRNA swarm are Dicer substrates as they consist of 25–27 nucleotide long siRNAs susceptible to cleavage by endogenous Dicer in humans and in mice.^2^

We have previously shown that siRNA swarms targeting a 653 base pair sequence of the essential viral *UL29* gene can inhibit active replication of circulating clinical HSV‐1 strains and acyclovir‐resistant strains in vitro, while being well tolerated by various cell types.^2^, ^4^, ^5^, ^6^, ^7^, ^8^ *UL29* has previously proven to be the most suitable target for anti‐HSV siRNA swarms. The gene encodes the essential protein ICP8, a single‐stranded DNA‐binding protein that is required throughout the replication cycle of HSV and is highly conserved.^4^, ^9^ The UL29‐targeted siRNA swarm has previously been proven efficacious as topical treatment in a corneal HSV infection model.^10^

HSV‐1 actively replicates in the orolabial epithelia and establishes latent infection in trigeminal ganglia (TG). Therefore, we wanted to study whether a single therapeutic dose of a UL29‐targeted siRNA swarm could protect mice from clinical disease and inhibit HSV infection in epithelia and the central nervous system (CNS). Intranasal siRNA delivery is attractive due to the possibility of delivering the siRNA to the brain via the olfactory bulb (OB) and thus bypassing the blood–brain barrier in mice.^11^ Intranasal administration of siRNA circumvents renal clearance, serum degradation by endonucleases, and prevents accumulation in nontarget tissues.^12^, ^13^ However, intranasal delivery is challenged by mucociliary clearance and active immune clearance.^14^ Furthermore, naked siRNA delivery to intracellular targets is challenging due to the negative charge of the siRNA, negating diffusion through cellular membranes. However, once in the cytoplasm, siRNA can remain active for up to a week.^15^ Accordingly, Chang et al. demonstrated the inhibition of SARS‐CoV‐2 in mice using pulmonary administration of naked anti‐SARS‐CoV‐2 siRNA and concluded delivery without nanocarriers to be superior over lipid and polymer systems.^16^

Modeling HSV‐1 infections in mice can be challenging but is possible via various routes of infection, one of which is intranasal infection. Intranasal infection with HSV‐1 can be considered translational and reproducible: Broberg et al. showed that intranasal inoculation can be used to establish acute HSV‐1 infections in the TG, also enabling the spread of viral DNA to the CNS.^17^ Furthermore, intranasal inoculation resulted in higher and more uniform titers in the TG than intralabial and corneal inoculation, proving intranasal delivery as the preferred route. Accordingly, Shivkumar et al. showed that intranasal inoculation was not only more potent than oral inoculation, but the infection in the TG also developed later into dermal infection without causing considerable neurological symptoms.^18^ Intranasally inoculated HSV spreads from the TG to the brain stem and cerebellum and from the OB to the temporal lobe, cerebral cortex and hippocampus in mice.^19^

Due to the attractiveness of intranasal delivery of siRNA treatments, and the translatability of intranasally inoculated HSV‐1 murine models, we wanted to investigate whether an intranasal dose of therapeutic UL29‐targeted siRNA swarm could protect against HSV‐1 infection in mice.

## 2 MATERIALS AND METHODS

**FIGURE 1:**
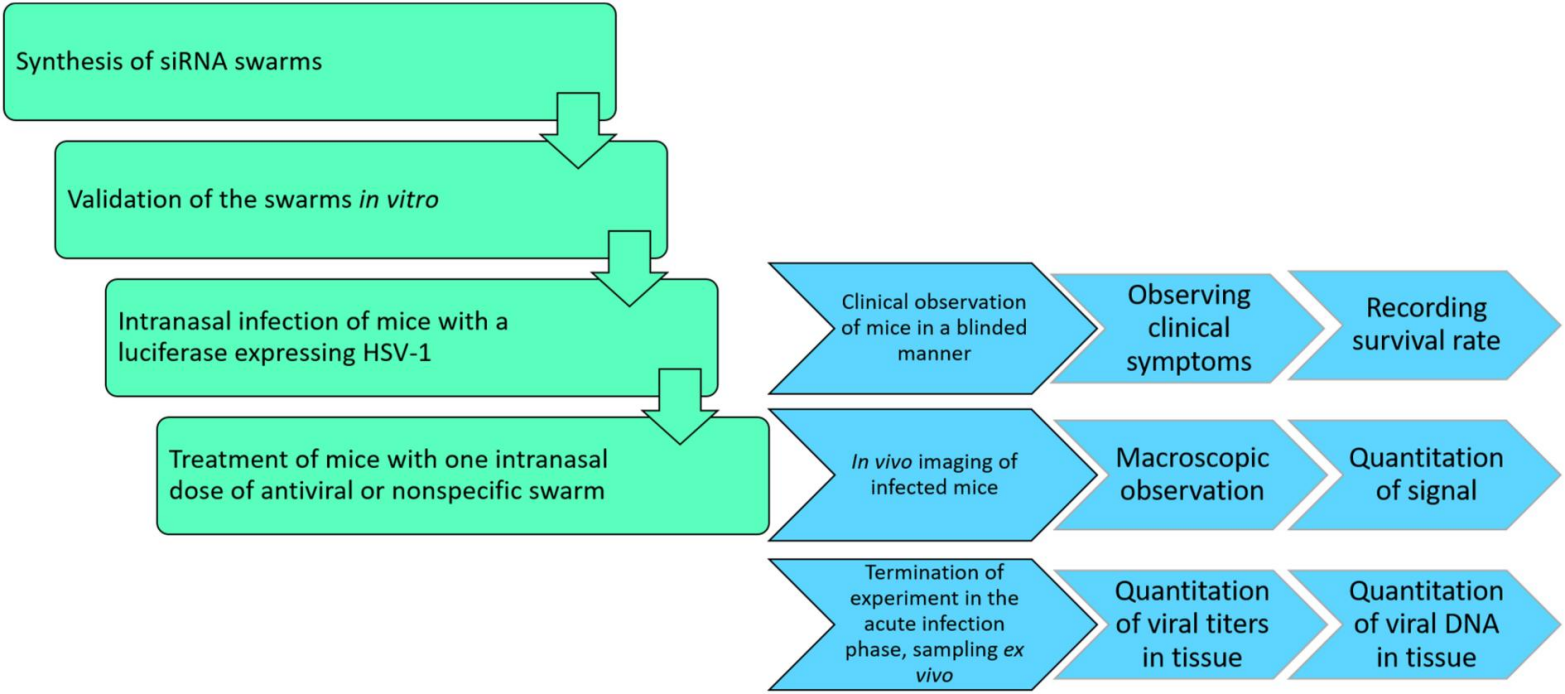
The experimental design of the study.

The experimental design of the study is illustrated in Figure 1.

### 2.1 Cells and viruses

Vero cells (African green monkey kidney cells, CCL‐81, ATCC), used for viral stock propagation and plaque assay, were cultured at 37°C and 5% CO_2_ in Dulbecco's modified Eagle medium (DMEM, Lonza) containing 7% heat inactivated fetal bovine serum (FBS) and 10 μg/mL gentamycin. A cell line representing cells of the nervous system, U373 MG, currently reclassified as U251 (HTB‐17, ATCC), was used for in vitro assessments of the siRNA swarms and was maintained in DMEM with 10% heat inactivated FBS and 10 μg/mL gentamycin.

HSV‐1(17+)Lox‐Luc (abbreviated here as HSV‐1‐LUC), containing a luciferase transgene under a human cytomegalovirus promoter,^20^ used in the animal model, was propagated in Vero cells and stored in 9% milk in water, as previously described.^10^, ^20^ In the in vitro assays, HSV‐1 (17+)Lox‐P_mCMV_GFP (abbreviated here as HSV‐1‐GFP) was used.^21^, ^22^ The virus was propagated and stored for in vitro antiviral assays as previously described.^2^

### 2.2 siRNA swarms and their assessment in vitro

The siRNA swarm that covers a 653 base pair sequence of the HSV‐1 UL29 gene was synthesized as previously described^2^ and purified using anion‐exchange chromatography.^23^ The swarm targets nucleotides 59302–59954 of HSV‐1 17+ (GenBank JN555585.1). A nonspecific control siRNA swarm derived from the *Escherichia coli* lac repressor lacI gene was used as a placebo treatment.^8^ An 88 base pair dsRNA, derived from the S‐segment of the bacteriophage phi6 genome, was used as a positive control for cytotoxicity in the cell viability assays.^24^

The batches of siRNA used in the experiments were assessed for antiviral efficacy and any toxicity in vitro. The lack of cellular toxicity of the batches was confirmed with a luminescent cell viability assay (CellTiter Glo [G7570, Promega]) at 48 h post transfection with 50 nM siRNA, as previously described.^2^ The assay confirming antiviral efficacy was performed in U373 MG cells as previously described.^2^, ^4^, ^5^, ^6^, ^8^ Shortly, the cells were transfected with 50 nM of siRNA and after 4 h were infected with HSV‐1‐GFP using 1000 plaque‐forming units (pfu) per well, corresponding to roughly 0.05 multiplicity of infection. For readout, the shed virus was quantified from the culture supernatant using the plaque assay.

### 2.3 Intranasal dosing of virus and siRNA

BALB/c mice, 4–6 weeks old, were used in this experiment (purchased from Janvier Labs, Le Genest‐Saint‐Isle, and housed at Central Animal Laboratory, University of Turku). The mouse strain and age during the experiment were chosen based on previous studies.^17^ The mice were maintained with food ad libitum in a 12 h dark–light cycle. On day zero, mice were weighed and divided into three groups of four mice each. Before infection, mice were given light anesthesia via an intraperitoneal injection using 1 mg/kg medetomidine (Cepetor Vet, 1 mg/mL, ScanVet)—75 mg/kg ketamine (Ketaminol vet, 50 mg/mL Intervet) mixture diluted in physiological NaCl solution. Mice were infected intranasally under anesthesia by slowly pipetting 25 μL phosphate‐buffered saline (PBS) containing 250,000 plaque forming units (pfu) of virus‐milk solution into both nostrils, allowing the mouse to inhale the solution. Therefore, the viral dose for each mouse was 5 × 10^5^ pfu. The optimal viral titer of HSV‐1‐LUC for this experiment was determined in preliminary studies (data not shown). After 5 min of incubation, the mice were awakened by a subcutaneous 1 mg/kg atipamezole hydrochloride (Antisedan, 5 mg/mL, Orion Pharma) injection into the flank.

At 4 h post infection, mice were anaesthetized again as aforementioned. The time point was chosen based on previous studies.^10^ Then, 25 μL of PBS solution containing 375 pmol of siRNA preparation was administered intranasally to each nostril (for a total of 750 pmol per mouse). Mock‐treated mice were administered 25 μL of PBS intranasally to each nostril. All treatments including the mock were blinded. Incubation and awakening were performed as above. The study was conducted under the permit ESAVI‐10570‐2019 of the national animal experiment board of Finland.

### 2.4 Follow‐up of mice

On days one to six post infection, mice were weighed and evaluated for visible clinical symptoms daily. The mice were either assigned as symptomatic (1) or symptomless (0). The monitored clinical symptoms included ruffled fur, hunched back, loss of fur, visible inflammation, as well as scared or sensitive behavior, or any other abnormal phenotype. If severe symptoms, such as lethargy or dehydration, associated with encephalitis, or more than 15% weight loss compared to day zero would have been observed, the mouse would be euthanized.

Localization of the HSV‐1 luciferase transgene, and the derived signal intensity, was monitored daily using the IVIS imaging system (IVIS spectrum, Perkin Elmer) after dosing 150 μL/20 g of intraperitoneal luciferin (XenoLight D‐Luciferin Potassium salt, PerkinElmer). Prior to luciferin injection, the mice were anaesthetized with isoflurane (Attane Vet, 1000 mg/g, VETMEDIC), with 2%–3% used for induction of anesthesia and 1%–2% for anesthesia upkeep during imaging. The mice were imaged in a 0° supine position, 90° side position, and 270° side position. Bioluminescence data was collected in the numerical format (photons/s) from the area of interest.

### 2.5 Tissue sampling

At day seven post infection, all mice were euthanized by CO_2_ asphyxiation (confirmed by heart puncture and blood withdrawal). Ex vivo tissue samples of the OBs, TG, brain, and nose were collected for subsequent virus titrations and quantitative real time PCR (qPCR). The ex vivo tissue samples were combined with DMEM containing 2% FBS, gentamycin, and amphotericin B. Five mm stainless steel beads (Qiagen) were added to the samples from the nose and TG, and 0.5 mm glass beads (Qiagen) were added to OB and brain samples. The samples were then shaken (50 Hz) for 3 min in TissueLyser LT (Qiagen), and the nose sample for an additional minute before being centrifuged and stored in −80°C.

### 2.6 Sample titration

The presence of infectious HSV‐1 in the ex vivo samples was analyzed by quantifying virus titers on Vero cells in 24‐well plates. The sample was combined with DMEM containing 2% FBS, gentamycin, and amphotericin B and titered in a series of 1:2, 1:10, and 1:50, or 1:2, 1:20, and 1:200. The cells were washed prior to infection. Subsequently, 300 µL of sample in medium was added on to the cells. The cells were incubated for an hour before washing and replacing the medium with DMEM‐containing 7% FBS, gentamycin, amphotericin B, and 80 mg/mL human immunoglobulin G (HyQvia, Takeda, MA, USA). After four days, the cells were fixed with methanol and stained with 0.5% crystal violet, and the plaques were counted from dried plates.

### 2.7 Quantitative real‐time PCR

The DNA extraction of the ex vivo samples were performed with E.Z.N.A. tissue DNA kit (Omega Bio‐tek) according to the manufacturer's protocol. qPCR for the HSV gene US6 (please see Ref.^25^ for primer sequences) was performed with the extracted DNA using SYBR green master mix (ThermoFisher Scientific) and Qiagen RotorGene U as previously described.^26^

### 2.8 Statistical analysis

SPSS Statistics 27.0.1.0 (IBM) software was used to perform all statistical tests. Pairwise comparisons were done with Mann–Whitney *U* test after homogeneity of variances was confirmed. A *p*‐value below 0.05 was considered significant. All graphs were created using ggplot2 library in *R*.^27^

## 3 RESULTS

### 3.1 In vitro results

**FIGURE 2:**
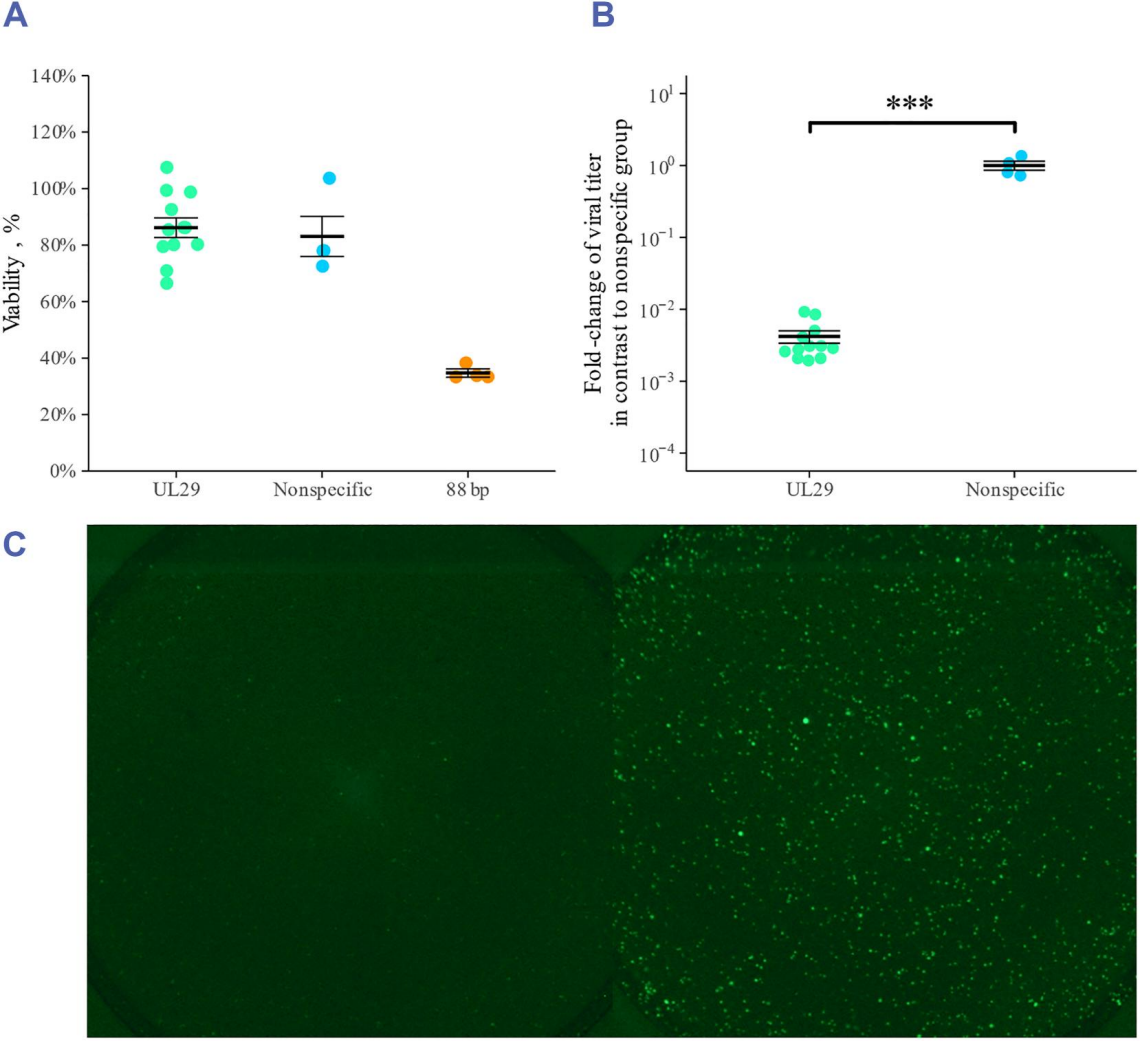
(A) Viability of U373 MG cells after siRNA swarm transfection. Cells were transfected with UL29‐targeted siRNA swarm, nonspecific siRNA swarm, transfection reagent only (mock) or a positive control for cytotoxicity, 88 base pair dsRNA. At 48 h post transfection, the viability of the cells was determined with a luminescent cell viability assay (CellTiter‐Glo, Promega). The data is shown as relative viability (%) in comparison to the mock‐treated group. (B) Antiviral efficacy of the UL29‐targeted siRNA swarm and the nonspecific siRNA swarm in U373 MG cells. U373 MG cells were transfected with the indicated siRNA swarms and 4 h later infected with 1000 plaque‐forming units (pfu) of HSV‐1‐GFP. At 48 h post infection, the supernatants were quantified for infectious virus by plaque titration on Vero cells. The titers are presented as fold change from the mean titer of the cells treated with the nonspecific siRNA swarm. In A and B, the whiskers represent the standard error to the mean and the thicker line in the middle indicates the mean. Pairwise comparisons were performed with the Mann–Whitney *U* test. Statistically significant differences are marked with asterisks (*p < 0.05; **p ≤ 0.01; ***p ≤ 0.001). (C) EVOS live fluorescent imaging of the antiviral treatment. The images present entire wells of 96‐well plate. The left image represents cells treated with antiviral siRNA swarms and the right image represents cells treated with a nonspecific siRNA swarm. The GFP signal is derived from HSV‐1‐GFP in infected cells.

Both the UL29‐targeted siRNA swarm and the nonspecific siRNA swarm were well tolerated by the U373 MG cells as the cellular viability was similar to that of the mock treatment (Figure 2A). In the same assay, the positive control for cytotoxicity, 88bp, significantly decreased cellular viability (Figure 2A). In the antiviral assay, the UL29‐targeted siRNA swarm significantly inhibited the replication of HSV‐1‐GFP in comparison to nonspecific siRNA treatment (*p* = 0.001) (Figure 2B). The GFP expressed by HSV‐1‐GFP infected cells was imaged (Figure 2C).

### 3.2 In vivo results

**FIGURE 3:**
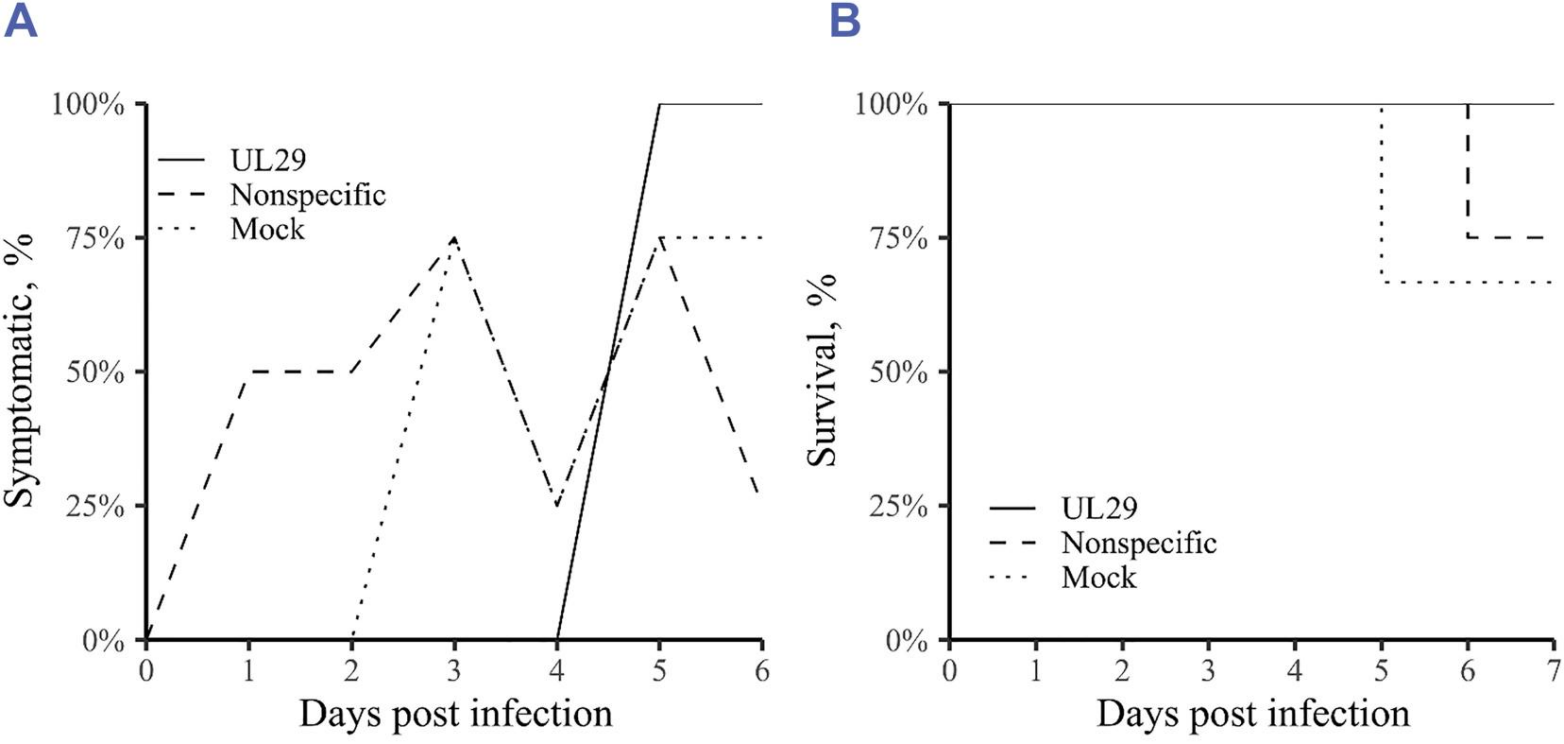
(A) Proportion (%) of mice in a group showing clinical symptoms of infection. Mice were infected intranasally with 5 × 10^5^ plaque‐forming units (pfu) of HSV‐1‐LUC. Four hours post infection, the mice were treated with 750 pmol of an antiviral UL29‐targeted siRNA swarm, 750 pmol of a nonspecific siRNA swarm, or with PBS only (mock). The mice were monitored daily for clinical symptoms, including ruffled fur, hunched back, loss of fur, visible inflammation, as well as abnormal, scared or sensitive behavior, or any other abnormal phenotype. (B) Survival of mice. If the mouse met the euthanasia criteria by severe symptoms (pre‐encephalitic) or by severe weight loss, it was considered not to have survived for the indicated day. If mice had survived and did not meet euthanasia criteria on day six, they were presumed to survive until day seven.

Placebo (nonspecific siRNA swarm) treated and mock (PBS) treated HSV‐1 infected mice displayed clinical symptoms beginning on day one and day three, respectively, with 75% of both groups symptomatic by day three (Figure 3A). The group treated with a UL29‐targeted siRNA swarm remained asymptomatic until day five (Figure 3A). Throughout the experiment, the only clinical symptom of the mice that was detectable by visual assessment, besides death or weight loss, was ruffled fur. At the end of the study, on day six, the UL29‐siRNA swarm‐treated group was the only group to have 100% of mice alive, whereas mice from both mock‐ and placebo‐treated groups were lost by day five and day six, respectively (Figure 3B). The weights of all three groups developed similarly throughout the experiment with a decreasing trend (data not shown).

**FIGURE 4:**
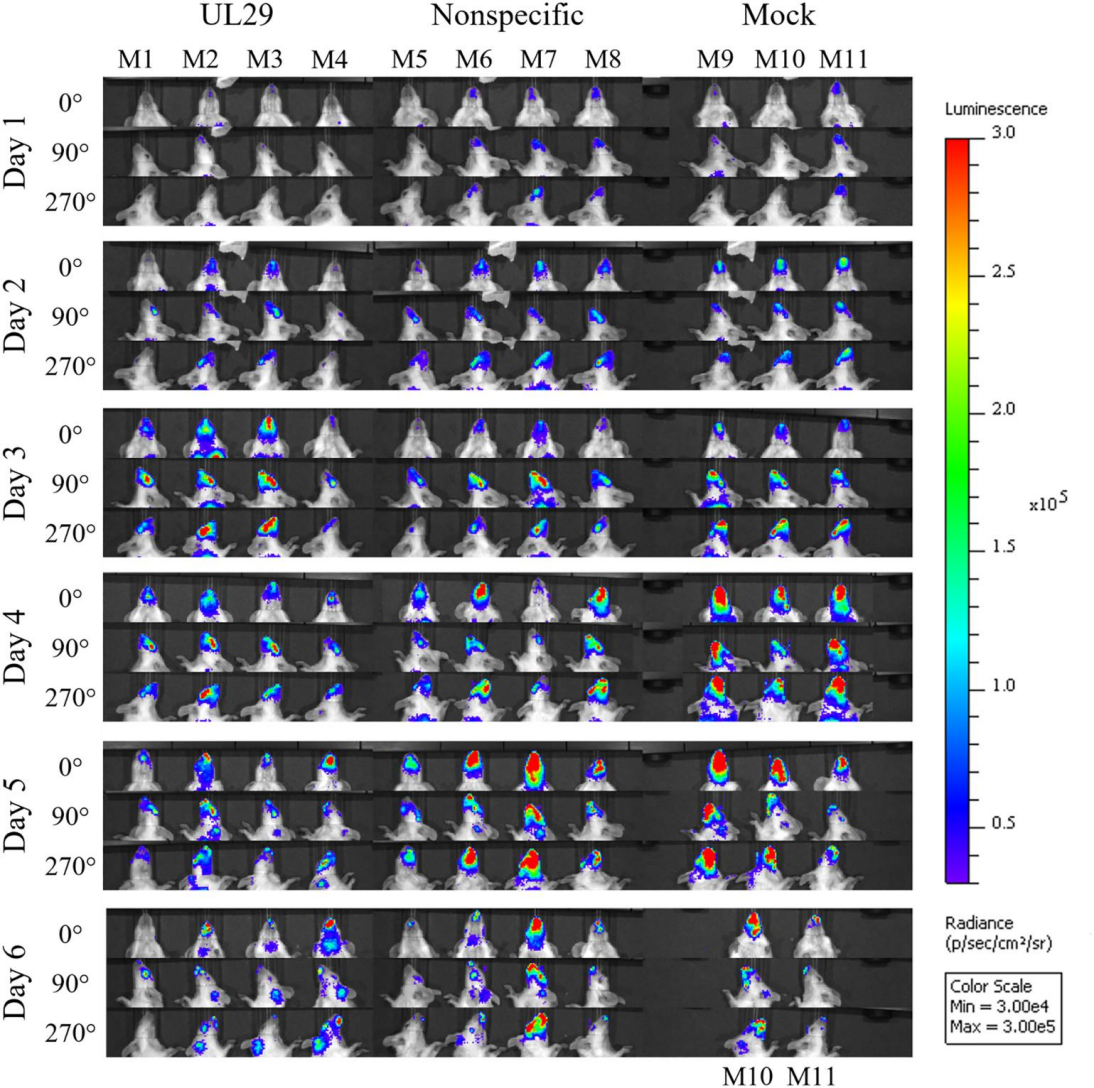
In vivo imaging of mice infected intranasally with HSV‐1‐LUC. All mice were infected intranasally with 5 × 10^5^ plaque‐forming units (pfu) of HSV‐1‐LUC. At 4 h post infection, they were treated with 750 pmol of an antiviral UL29‐targeted siRNA swarm, 750 pmol of a nonspecific siRNA swarm, or with PBS only (mock). All mice were imaged daily using IVIS Spectrum. The luminescent signal is emitted from the reaction between the injected luciferin and the luciferase transgene of HSV‐1‐LUC. The radiance color scale is shown on the right. For each day, the mice were imaged in a 0° supine position (first row), 90° side position (second row), and 270° side position (third row). All images are on the same scale and were taken with a 20 s exposure time. Mice M1–M4 were treated with a UL29‐targeted siRNA swarm, mice M5–M8 were treated with a nonspecific siRNA swarm, and mice M9–M11 received PBS as a mock treatment.

**FIGURE 5:**
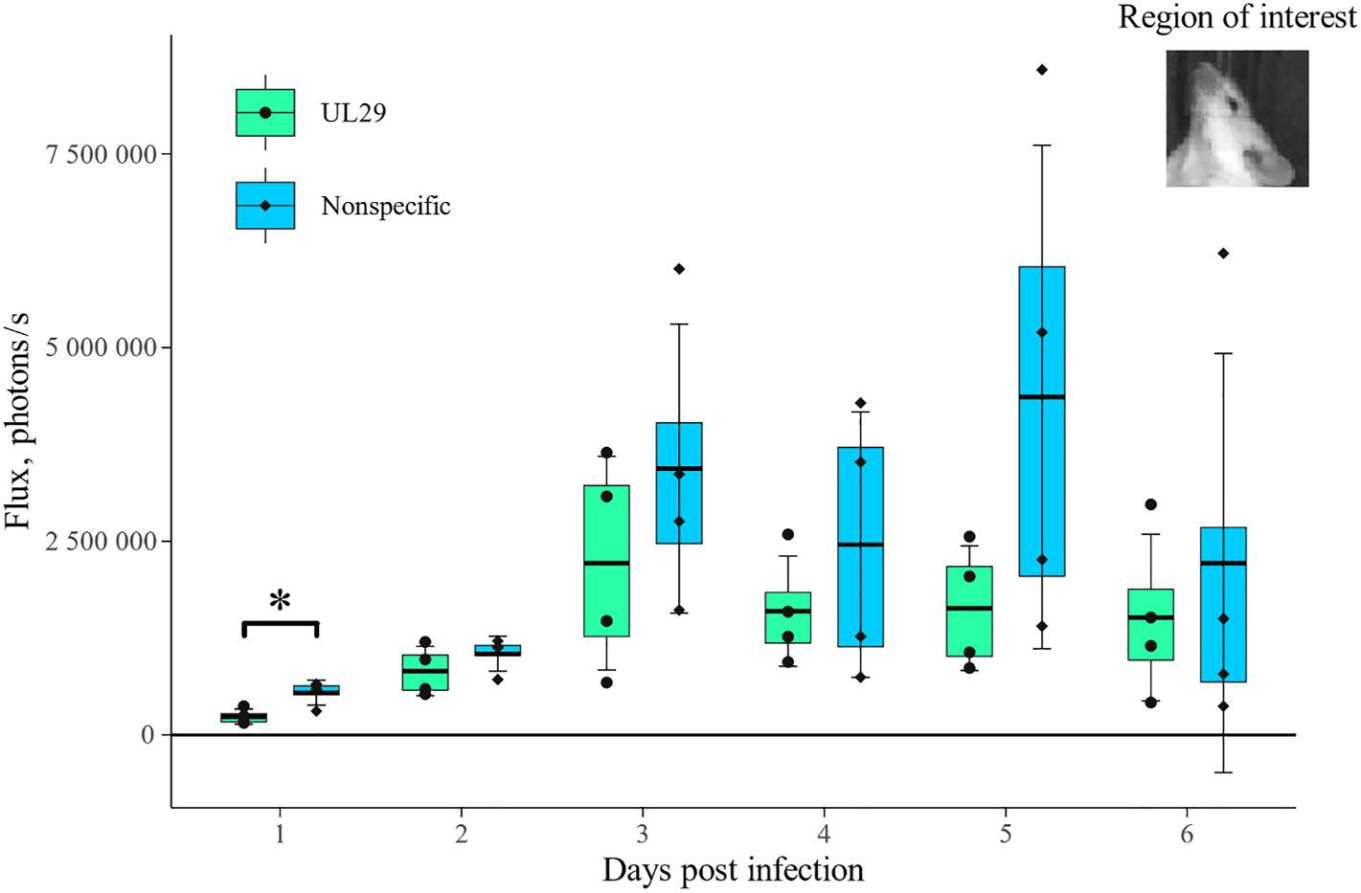
Quantitation of luminescence radiance from the heads of the HSV‐1‐LUC‐infected mice. Mice, which were infected intranasally with 5 × 10^5^ plaque‐forming units (pfu) of a luciferase‐expressing HSV‐1, HSV‐1‐LUC, were treated 4 h post infection with an intranasal 750 pmol dose of an antiviral UL29‐targeted siRNA swarm or a nonspecific siRNA swarm. The mice were imaged with IVIS daily for six consecutive days, and the derived signal was quantified. The region of interest for the signal quantification was similar for each mouse, covering the whole head, as clarified in the inscribed image. For each mouse, the radiance was calculated as a mean of measurements from all three head positions presented in Figure 2. Statistically significant differences (*p* < 0.05) are marked with an asterisk (*). Boxplot shows first quartile and third quartile. Bold horizontal line shows mean and whiskers show standard deviation to mean.

The mice infected intranasally with HSV‐1‐LUC were imaged for six consecutive days after infection (D1–D6). The signal from luciferase HSV‐1‐LUC was detected in all mice already on day one. The signal developed similarly within each group: At first, the signal centralized in the perinasal area, spreading to surrounding head areas, reaching peak intensity on day five (Figure 4). In contrast to the UL29‐siRNA swarm‐treated group, the placebo treatment allowed the development of higher luminescence levels (Figure 5). On day one, the luminescence signal from the head region of the mouse (for region of interest, equal for each mouse, please see Figure 5 inscribed image) was significantly lower (*p* = 0.043) in the group treated with a UL29‐targeted siRNA swarm than in the placebo‐treated group (Figure 5). Whether the infection is epithelial or neural cannot be directly concluded from the images.

**FIGURE 6:**
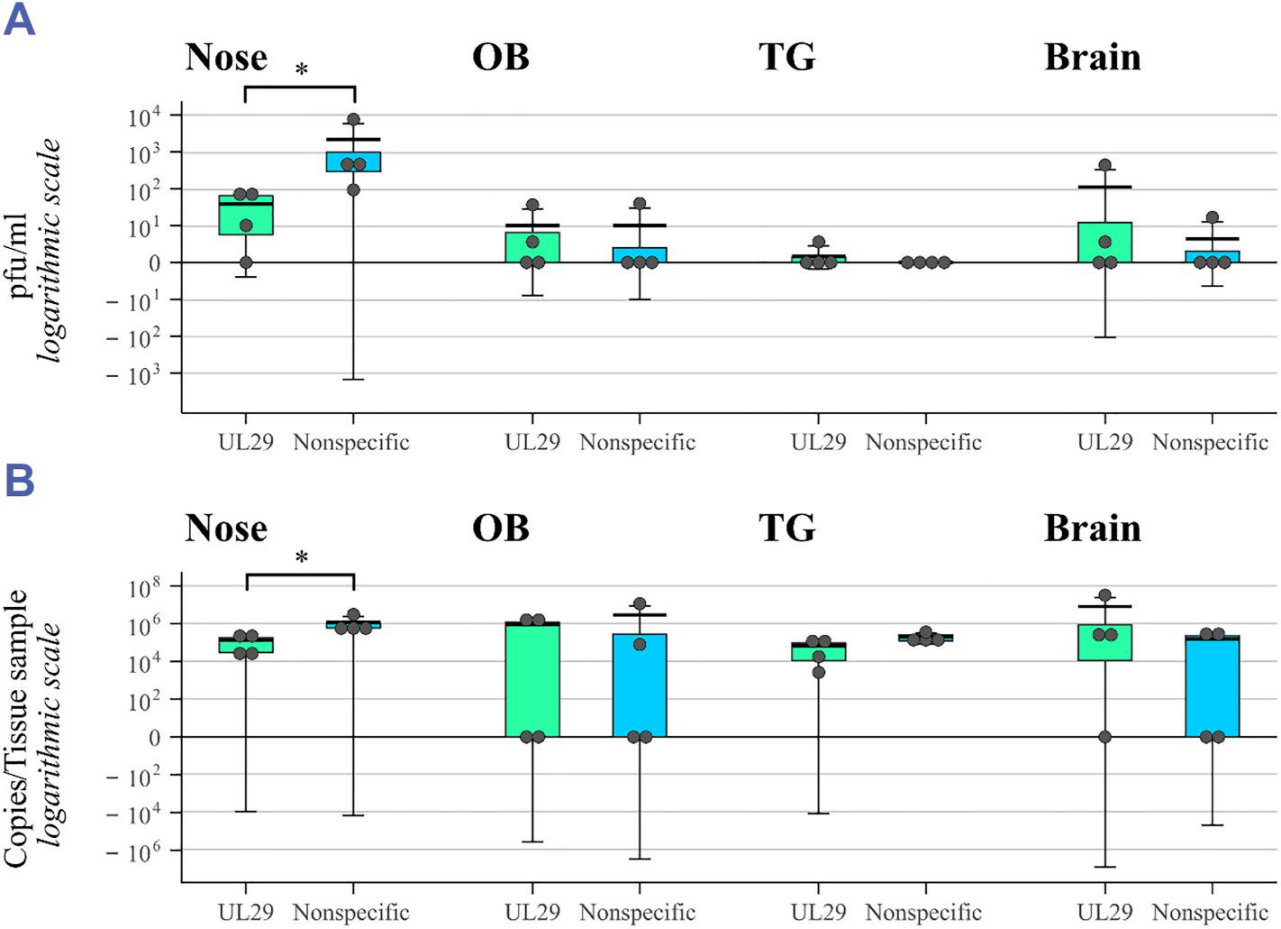
Presence of HSV in tissue samples of HSV‐infected mice treated with a UL29‐siRNA swarm or a nonspecific siRNA swarm (placebo). (A) Viral titers of HSV in ex vivo tissue samples. (B) Number of HSV DNA copies in ex vivo tissue samples. Statistically significant differences (*p* < 0.05) are marked with an asterisk (*), other differences were nonsignificant. Boxplot shows first quartile and third quartile. Bold horizontal line shows mean and whiskers show standard deviation to the mean. HSV, herpes simplex viruses; OB, olfactory bulb; pfu, plaque‐forming unit; TG, trigeminal ganglion.

Tissue samples of nasal epithelium (“nose”), OB, TG, and brain were collected from all mice upon euthanasia. The samples were quantified for infectious HSV‐1 by a plaque assay and for HSV DNA copies via qPCR. The nose samples were all positive for HSV DNA and for HSV‐1 cultures, except for one UL29‐targeted siRNA swarm‐treated mouse (M4), which had a negative viral culture (Figure 6). Both the viral titers and the amount of HSV DNA copies of the nose samples were significantly higher (*p* = 0.029) in the placebo‐treated group than in the UL29 siRNA swarm‐treated group (Figure 6). The OB, TG, and brain samples were mostly similar between the groups, with roughly half of the mice positive in both groups (Figure 6). The titers of OBs, TGs, and brain were also equal among the groups (Figure 6A). Likewise, the HSV DNA amounts of OBs and brain were similar in the groups (Figure 6B). For TG, the HSV DNA amounts in the UL29 siRNA swarm‐treated group were inferior to the placebo‐treated group, but the difference was statistically insignificant (*p* = 0.083) (Figure 6B).

## 4 DISCUSSION

The intranasal administration route is attractive for antiviral RNA interference (RNAi) due to its noninvasiveness, ease of access, and ability to bypass clearance. To our knowledge, this is the first published study administering antiviral siRNA intranasally to treat HSV‐1 infections. In this study, we assessed the effect of a single intranasal therapeutic dose of a UL29‐targeted siRNA swarm in an intranasal model of HSV‐1 infection. A nonspecific siRNA swarm was used as a placebo. Prior to the in vivo study, we confirmed the antiviral efficacy and the lack of cytotoxicity of the used siRNA swarms (Figure 2). Both the UL29‐targeted antiviral siRNA swarm and the nonspecific siRNA swarm were well tolerated (Figure 2A). Furthermore, the UL29 siRNA swarm exhibited proper antiviral efficacy in comparison to the nonspecific siRNA swarm (*p* = 0.001), preventing over 99% of HSV‐1 replication (Figure 2B). These results are in line with our previous research.^2^, ^4^, ^5^, ^6^, ^7^, ^8^

In mice, the anti‐HSV UL29‐targeted siRNA swarm treatment protected them from the onset of symptoms. The mice treated with the UL29 siRNA swarm developed symptoms as late as on day five, whereas 75% of placebo‐treated mice were already symptomatic at that time, with symptoms developing already from day one (Figure 3A). Therefore, the intranasal dose of UL29 siRNA swarm protected the mice from clinical signs of disease. This result is in line with previous research, where a topical siRNA swarm treatment to the infected cornea decreased symptoms and subsequently increased the survival rate of mice.^10^ Also in this study, the UL29 siRNA swarm also increased the survival rate by 25% (Figure 3B), but given the small group size, our result on increased survival should not be considered significant before subsequent, larger experiment series or an experiment with a higher dose of virus. The treatment did not affect the weight development of the mice (data not shown).

The signal derived from the activity of the luciferase transgene of the HSV‐1‐LUC strain revealed that the infection spread equally within each group within a similar timeframe (Figure 4). Most likely the infection spread to the CNS via OBs as suggested by the literature.^19^ The later onset of the symptoms in the UL29‐targeted siRNA swarm treated group was reflected by the lower luminescence in the UL29‐targeted siRNA swarm‐treated group compared to the placebo‐treated group during the first five days of the study (Figures 4 and 5). However, the difference in the luminescence intensity between the two groups was significant only on day one (*p* = 0.043) (Figure 5). Likely, this protecting effect of the UL29‐targeted siRNA swarm, detected in the luminescence intensity on day one (Figures 4 and 5), is why the mice in the UL29 siRNA swarm‐treated group did not exhibit detectable symptoms until day five, whereas the placebo‐treated mice were symptomatic from the start of the study (Figure 3A). Furthermore, the UL29 siRNA swarm treatment slowed the progress of the infection, as on day two the signal from UL29 siRNA swarm‐treated mice, M1 and M4, had not developed to orofacial or CNS infection, although a slight luminescence signal could be detected (Figure 4). This contrasts with the placebo‐treated group, where the signal had already progressed toward CNS (Figure 3A). The lack of CNS symptoms, despite the probable presence of HSV‐1 in the CNS (Figures 4 and 6) is likely due to the attenuated phenotype of HSV‐1‐LUC^10^ or the viral dose, which was selected to allow the accomplishment of the experiment without unnecessary loss of mice (data not shown).

The anti‐HSV UL29‐targeted siRNA swarm decreased the local viral replication and viral load in nose tissue samples in comparison to the nonspecific siRNA swarm as was observed by both plaque assay (*p* = 0.029) and qPCR (*p* = 0.029), respectively (Figure 6). Therefore, similar to topical treatment to the eye,^10^ also an intranasal dose of a UL29‐targeted siRNA swarm can inhibit local HSV‐1 replication and decrease the viral load in mice (Figure 6). Nevertheless, more extensive in vivo studies are required to assess whether repeated therapeutic dosing, common for antiviral treatment, could improve the detected antiviral outcome.

The treatment of mice with the UL29‐targeted siRNA swarm decreased the HSV‐1‐LUC transgene expression on day one (Figure 5), suggesting that the treatment either decreased the early viral load and/or delayed the spread of the virus to the CNS, and thus delaying the onset symptoms. However, the results from the TG, OB, and brain tissues collected on day six did not show a significant difference in viral titers or DNA copy numbers between the treatment groups (Figure 6) as the viral titers in all CNS samples were low in most infected mice. In order to study the role of antiviral therapy to HSV‐1 in the nervous system in more detail and more reliably, the in vivo experiment would have needed to be longer to allow the establishment of latency and reactivation of any latent virus either in vivo or ex vivo via an explant culture. Here, in scope of this research, it was not feasible, but is an important research question for future experiments. The antiviral UL29 siRNA swarm is not by any means a candidate to cure latency, but finding out whether it might have a role in decreasing the amount of virus establishing latency, or in prevention of reactivation, would be of high interest. Nevertheless, in this study, the UL29‐targeted antiviral siRNA swarm treatment showed a tendency toward decreasing the number of viral DNA copies in TG (*p* = 0.083) in comparison to nonspecific siRNA swarm (Figure 6B). Thus, it could be speculated that the treatment might affect the latent viral load.

The results derived from the intranasal in vivo infection results indicate high promise for the antiviral siRNA swarm, even without any delivery reagent. In preliminary studies, we also investigated intranasal delivery of the siRNA swarm encapsulated in commercial *in vivo*‐JetPEI transfection reagent (Polyplus), but it did not improve treatment outcome (data not shown). As previously reported by Ferrari et al., PEI becomes immobilized in mucus due to its positive charge.^28^ Thus, similar to Chang et al., we also found naked siRNA to be more optimal when administered intranasally.^16^ Prophylactic dosage, multiple dosing, or siRNA encapsulation in mucoadhesive or mucopenetrating nanocarriers, that enable endosomal escape, could improve the efficacy of our siRNA therapy.

As a conclusion, a single therapeutic dose of an intranasally administered UL29‐targeted siRNA swarm protects HSV‐1‐infected mice from the onset of clinical symptoms (Figure 3A) significantly prevents local viral replication and spread (Figures 4 and 5) as well as significantly decreases the viral load (Figures 4, 5, 6). Next, to uncover the extent of the antiviral efficacy of the UL29‐targeted siRNA swarm, repeated dosing and a careful choice of delivery agents should be assessed in more extensive in vivo studies. Nevertheless, these results, although derived from a limited number of mice, are valuable as they confirm the in vivo efficacy of siRNA swarms in the treatment of HSV‐1 infection as well as emphasize the potential of the siRNA swarm approach as an intranasal treatment for applications also beyond HSV‐1 infection.

## AUTHOR CONTRIBUTIONS

Conceptualization, Kiira Kalke, Veijo Hukkanen; Methodology, Tuomas Lasanen, Fanny Frejborg, Liisa M. Lund, Marie C. Nyman, Julius Orpana, Huda Habib, Salla Alaollitervo, Alesia A. Levanova, Minna M. Poranen, Kiira Kalke, Veijo Hukkanen; Formal analysis, Tuomas Lasanen, Liisa M. Lund, Kiira Kalke; Investigation, Tuomas Lasanen, Fanny Frejborg, Liisa M. Lund, Marie C. Nyman, Julius Orpana, Huda Habib, Salla Alaollitervo, Kiira Kalke, Veijo Hukkanen; Resources, Minna M. Poranen, Veijo Hukkanen; Data curation, Tuomas Lasanen, Liisa M. Lund, Kiira Kalke; Writing – original draft preparation, Tuomas Lasanen, Fanny Frejborg; Writing – review and editing, Tuomas Lasanen, Fanny Frejborg, Liisa M. Lund, Marie C. Nyman, Julius Orpana, Huda Habib, Salla Alaollitervo, Alesia A. Levanova, Minna M. Poranen, Kiira Kalke, Veijo Hukkanen; Visualization, Tuomas Lasanen, Fanny Frejborg, Kiira Kalke; Supervision, Minna M. Poranen, Kiira Kalke and Veijo Hukkanen; Project administration, Minna M. Poranen, Veijo Hukkanen; Funding acquisition, Minna M. Poranen, Veijo Hukkanen. All authors have read and agreed to the published version of the manuscript.

## CONFLICT OF INTEREST STATEMENT

The authors declare no conflict of interest.

## ETHICS STATEMENT

The study was conducted under the permit ESAVI‐10570‐2019 of the national animal experiment board of Finland. The recombinant virus use was according to the notification 018/M/2018 of the Board of Gene Technology.
